# 1025. Consequences of Low-level Viremia Among Women with HIV in the United States

**DOI:** 10.1093/ofid/ofad500.056

**Published:** 2023-11-27

**Authors:** Amalia Aldredge, Cyra Christina Mehta, Lauren F Collins, Cecile D Lahiri, Maria L L Alcaide, Jack DeHovitz, Kathryn Anastos, Michael Plankey, Jodie A Dionne, Michelle Floris-Moore, Michael F Schneider, Audrey L French, Phyllis C Tien, Anandi N Sheth

**Affiliations:** Emory University, Atlanta, GA; Emory University School of Medicine, Atlanta, Georgia; Emory University School of Medicine, Division of Infectious Diseases, Atlanta, Georgia; Emory University, Atlanta, GA; Division of Infectious Diseases, Department of Medicine, University of Miami Miller School of Medicine, Miami, Florida; State University of New York-Downstate Medical Center, New York, New York; Department of Medicine, Albert Einstein College of Medicine, Bronx, New York, Bronx, NY; Georgetown University, Washington, District of Columbia; University of Alabama at Birmingham, Birmingham, AL; Department of Epidemiology, Johns Hopkins Bloomberg School of Public Health, Baltimore, Maryland; Stroger Hospital of Cook County, Chicago, Illinois; Division of Infectious Diseases, Department of Medicine, University of California, San Francisco, San Francisco, California and Medical Service, Department of Veterans Affairs, San Francisco, California, San Francisco, CA; Emory University School of Medicine, Atlanta, Georgia

## Abstract

**Background:**

Low-level viremia (LLV) is common in people with HIV taking antiretroviral therapy (ART), and has been associated with virologic failure, resistance, and may increase the risk of non-AIDS comorbidities (NACM). However, despite sex-differential HIV pathogenesis, treatment response, and NACM risk, women have been underrepresented in prior studies.

**Figure:**
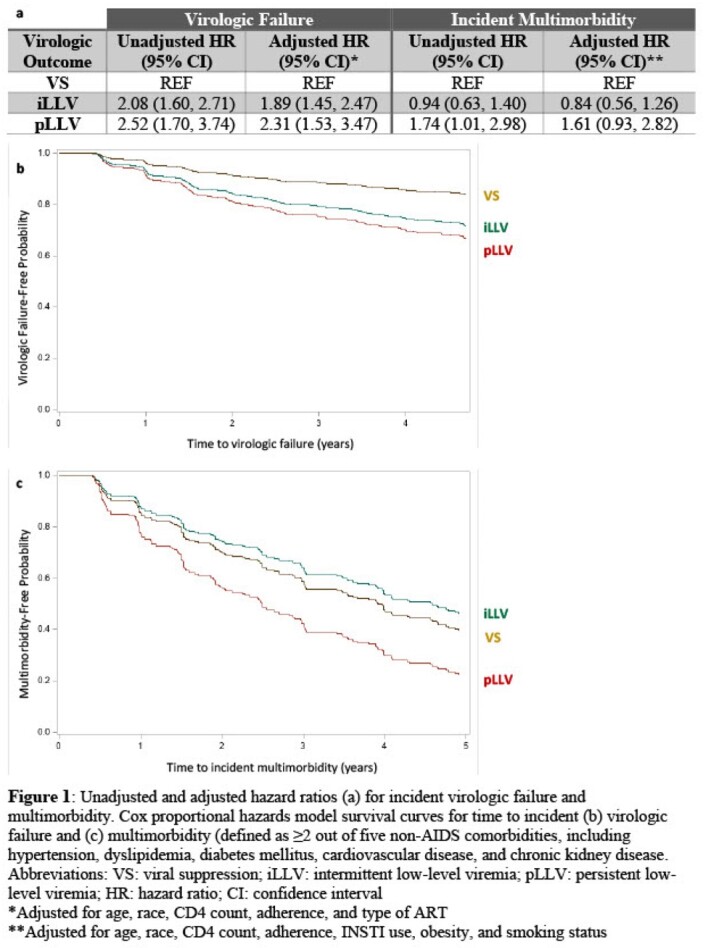

**Methods:**

We included Women’s Interagency HIV Study (WIHS) participants from 2003-2020 receiving ART for ≥1 year with ≥2 consecutive HIV-1 viral loads (VL) < 200 c/mL. Women were then categorized using 4 consecutive VL measurements (virologic categorization period) and then followed for 5 years for outcomes assessment. At end of the virologic categorization period, women were categorized as: virologic suppression (VS; all VL undetectable), intermittent LLV (iLLV; non-consecutive detectable VL up to 199 c/mL), persistent LLV (pLLV; ≥2 consecutive detectable VL up to 199 c/mL), or virologic failure (VF; any VL ≥200 c/mL). Adjusted Cox proportional hazards models were used to estimate the association of virologic category with time to incident a) virologic failure, and b) multimorbidity (defined as ≥2 of 5 NACM [hypertension, dyslipidemia, diabetes, cardiovascular disease, kidney disease]).

**Results:**

Of 1,598 participants, 275 (17%) had VF during virologic categorization period and were not included in analyses. Median age of included women was 47 years, 64% were Black, 21% were Hispanic, median CD4 652 cells/µL, and 89% reported ≥95% ART adherence. VS, iLLV, and pLLV occurred over a median of 18 months in 58%, 19%, and 6% respectively. Compared to VS, the adjusted hazard ratio (aHR) for incident virologic failure was 1.89 (95% CI 1.45-2.47) for iLLV and 2.31 (1.53-3.47) for pLLV. After excluding 543 women with baseline multimorbidity, aHR for incident multimorbidity was 0.84 (0.56-1.26) for iLLV and 1.61 (0.93-2.82) for pLLV (Figure 1).

**Conclusion:**

iLLV or pLLV occurred in 25% of WIHS participants. Compared to women with VS, iLLV and pLLV were associated with increased virologic failure risk, whereas only pLLV was associated with increased multimorbidity risk, though the association did not persist after covariate adjustment. Research is needed to assess the effect of sex on LLV prevalence and consequences to inform clinical management of LLV.

**Disclosures:**

**Cyra Christina Mehta, PhD, MSPH**, Merck: Grant/Research Support **Lauren F. Collins, MD, MSc**, Curio Science: Honoraria **Cecile D. Lahiri, MD, MS**, Merck: Grant/Research Support|Theratechnologies, LLC: Advisor/Consultant|Theratechnologies, LLC: Honoraria **Maria L L. Alcaide, MD**, Discidium Biosciences: Board Member|Gilead: Honoraria|Merk & Co: Honoraria|Senhwa Biosciences: Honoraria|Virology Education: Honoraria **Michelle Floris-Moore**, ViiV Healthcare: Advisor/Consultant **Phyllis C. Tien, MD, MSc**, Merck: Grant/Research Support

